# A Microbial Phenomics Approach to Determine Metabolic Signatures to Enhance Seabream *Sparus aurata* Traceability, Differentiating between Wild-Caught and Farmed

**DOI:** 10.3390/foods13172726

**Published:** 2024-08-28

**Authors:** Marta Nerini, Alessandro Russo, Francesca Decorosi, Niccolò Meriggi, Carlo Viti, Duccio Cavalieri, Massimiliano Marvasi

**Affiliations:** 1 Department of Biology, University of Florence, Via Madonna del Piano, 50019 Firenze, Italy; marta.nerini@unifi.it (M.N.); alessandro.russo@unifi.it (A.R.); duccio.cavalieri@unifi.it (D.C.); 2 Department of Agriculture, Food, Environment and Forestry (DAGRI), University of Florence, 50019 Florence, Italy; francesca.decorosi@unifi.it (F.D.); carlo.viti@unifi.it (C.V.); 3 Institute of Agricultural Biology and Biotechnology (IBBA), National Research Council (CNR), 56124 Pisa, Italy; niccolo.meriggi@cnr.it

**Keywords:** traceability, fish, food microbiology, food quality, EcoPlate

## Abstract

Background: The need for efficient and simplified techniques for seafood traceability is growing. This study proposes the Biolog EcoPlate assay as an innovative method for assessing wild and farmed *Sparus aurata* traceability, offering advantages over other molecular techniques in terms of technical simplicity. Methods: The Biolog EcoPlate assay, known for its high-throughput capabilities in microbial ecology, was utilized to evaluate the functional diversity of microbial communities from various organs of *S. aurata* (seabream) from the Mediterranean area. Samples were taken from the anterior and posterior gut, cloaca swabs and gills to distinguish between farmed and wild-caught individuals. The analysis focused on color development in OmniLog Units for specific carbon sources at 48 h. Results: Gills provided the most accurate clusterization of sample origin. The assay monitored the development of color for carbon sources such as α-cyclodextrin, D-cellobiose, glycogen, α-D-lactose, L-threonine and L-phenylalanine. A mock experiment using principal component analysis (PCA) successfully identified the origin of a blind sample. Shannon and Simpson indexes were used to statistically assess the diversity, reflecting the clusterization of different organ samples; Conclusions: The Biolog EcoPlate assay proves to be a quick, cost-effective method for discriminate *S. aurata* traceability (wild vs. farmed), demonstrating reliable reproducibility and effective differentiation between farmed and wild-caught seabream.

## 1. Introduction

Authentication and food fraud detection is part of the complex network of actions that include food traceability. Traceability is a process which involves several different disciplines, including information, logistics, risk management, quality and safety [1]. The determination of food origin holds significant importance, not just for ensuring safety and quality, but also for impacting customer satisfaction and the price consumers are willing to pay. Extensive surveys conducted among European citizens have consistently shown that purchasing decisions regarding seafood products are influenced by factors such as product origin and the methods employed in fishing or farming practices [2]. Thus, the interplay between authenticity, brand recognition and consumers’ trust plays a vital role in supporting their purchasing choices. In many cases, legal requirements are introduced to certify the specific origin of food [3] and EU Regulation 2018/274, including to prevent allergenic or toxic molecules. To that end, governments, international standardization and non-governmental certification organizations play major roles in protecting food authenticity.

The globalization of the seafood trade and the lack of standards for information exchange have made tracking and tracing seafood very challenging [4]. In the supply chain and the seafood industry, there is a need for improved traceability, sustainability and food safety.

From technical perspectives, food authenticity is protected by employing a number of techniques, ranging from chemical analysis, isotope, and biogeochemical targets, to metagenomics [5,6]. The use of microbial metagenomics has attracted attention to traceability. The forensic aspect of the use of microbial communities as a signature for a specific environment has been shown in different habitats, showing that the generation of a typical microbial community is not stochastic, but finely regulated and reproducible [7]. Microbial communities in the environment have specific signatures that are impossible or very difficult to reproduce in vitro leading to possible fraud. This is true not only with reference to taxonomical populations, including evenness and richness, but also for metabolic traits, which include particular or unique genes and metabolic pathways (biomarkers) [8]. Based on this evidence, microbial metagenomics has been proposed as a tool to associate specific signatures.

Microbial communities can therefore contribute to food fraud detection [9] by (1) authenticating the origin of a product in terms of geographical or botanical/animal provenance or organs; (2) proving the absence of adulteration. In this scenario, the use of phenomics (Biolog EcoPlate) as a possible alternative to help in identifying where fish came from is tested in this work. EcoPlate Biolog is a type of high-throughput microbial ecology assay that is used to measure the functional diversity of microbial communities and monitor various environmental samples [10,11,12,13]. A particular biological system, whether ecosystem or microcosm, depends on the interplay of three factors—environment, biological community structure (diversity) and biological activity (function) [14]. Therefore, the final outcome in the phenotype microarray results from this unique interaction. Typically, the EcoPlate assay involves the use of a 96-well microplate that contains a set of 31 carbon sources (and one negative control) repeated three times, which are used as sole carbon and energy sources by different microbial functional groups. By measuring the utilization of these carbon sources by the microbial community, we aim to unravel differences in the microbial communities accordingly, not only with the microbiome associated with wild or farmed fish, but also with the organ from which the microbial community is isolated. EcoPlates are particularly useful for identifying the functional capabilities of microbial communities, which can inform our understanding of ecosystem processes and help us to develop strategies for managing traceability.

## 2. Materials and Methods

### 2.1. Fish Material and Dissections

The study area was chosen to be the Costa degli Etruschi, extending from Livorno to Piombino (Italy). This region is notable for its production of seabream, both wild and farmed, which is of substantial economic value. This focus aligns with the area’s prominence in the seafood industry [15]. Wild-caught seabream (*Sparus aurata*) and farmed seabreams were purchased by a local high-quality and ISO certified seafood retailer (certified with UNI EN ISO 9001:2015 and ISO 25012:2014) with certified and labeled origin of fishing, farming and date of delivery. All seabream used in this study, both wild-caught and sea-farmed, came from a very limited area ensuring that samples were obtained in close proximity to each other, specifically in a few square kilometers from the coast of Tuscany, Italy, within the FAO fishing area 37.1.3 [16]. Due to the difficulty of obtaining matched fish in the same area, in particular with reference to the wild-caught individuals, experiments were conducted on different days. A total of 46 specimens were analyzed. The specimens comprehended gills, cloaca, anterior and posterior gut obtained from 23 individuals purchased at two different times, in April 2023 (*n* = 9) and February 2024 (*n* = 14) to average season differences: 10 for wild-caught seabreams, 10 for farmed seabreams and 3 for the mock experiment. Preliminary experiments showed that gut samples were unsuitable for the analyses. For this reason, anterior and posterior gut were not sampled for the rest of the individuals. The average fish weight was 671 ± 46 g for wild-caught seabreams and 411 ± 22 g for farmed seabreams. The age of the fish was unknown.

### 2.2. Sample Preparation for EcoPlate Assay

**Figure 1:**
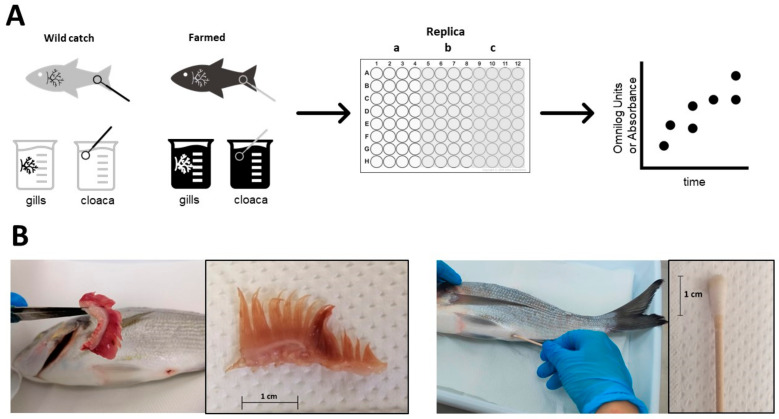
Overview of experiments designed to use 96-well EcoPlate to assess traceability (see the Section 2 for details). (**A**) Briefly, wild-caught and farmed samples were obtained and 1 g of gills was resuspended in water. Similarly, a swab was used to remove fecal material from the cloaca. The suspensions were aliquoted in the 96-well EcoPlate, which was divided into three technical replicas (represented by lowercase letter and different shadows of the 96-well plate). (**B**) Example of extraction of gills and sampling in the cloaca.

The main overview of the process is described in Figure 1. Fish were processed within 30 min of purchase and within 6 h of fishing. All fish were delivered on ice in refrigerated trucks. From each fish, four different specimens were collected: a cloacal swab, a fragment of gill, the anterior gut and the posterior gut. A fragment of 1 g of the left gill was collected using a pair of scissors previously sterilized on flame (Figure 1A). The cloaca was sampled inserting the collection tip of a sterile swab (for a depth of about 1.5 cm) inside and slowly rotating the swab against the gut wall for 4 rotations. The swab (cotton) portion was therefore cut with a scissor previously sterilized on the flame each time. Afterwards, the fish was dissected to recover the entire gut using a sterile scalpel; the gut was then divided into two portions, identified as the anterior and posterior gut. From those sections 1 g of anterior and posterior gut was cut. All samples were resuspended in 10 mL of sterile demineralized water and vortexed with a Vortex-Genie 2T (Scientific Industries, New York, NY, USA) for 10 min to help resuspend the microorganisms in the water. After vortexing, 100 µL of the swab was directly plated in the EcoPlate. Gill suspensions were diluted to 1:10, then 100 µL were plated in the EcoPlate [10,11,12,13]. For gut samples, different dilutions were tested (1:2, 1:10, 1:100) due to the difference in turbidity caused by the stochastic presence of the gut contents, then 100 µL of each dilution was plated in the EcoPlate. EcoPlates were incubated at 25 °C inside an OmniLog Reader (Biolog) and monitored automatically every 15 min for color changes in the wells for 48 h. Raw kinetic data (Arbitrary OmiLog Units) were retrieved using the OmniLog, OL_PM_FM/Kin 1.30, File Management/Kinetic Plot software (Version 1.7) (Biolog, Hayward, CA, USA). After 2 days of incubation, EcoPlates were collected and stored at −20 °C.

### 2.3. Statistical Analysis

All statistical analyses were carried out using the GraphPad Prism 9.2.0 package. Differences in Arbitrary OmniLog Units (AOU) of selected metabolites developed at 0 h, 24 h and 48 h between farmed and wild-caught samples. We employed the Kruskall–Wallis tests for comparing groups. Principal Component Analysis (PCA) was performed using the prcomp function to depict sample distribution according to the different substrate consumption profiles (D,L-α-Glycerol Phosphate, D-Cellobiose, Glucose-1- Phosphate, Glycogen, i-Erythritol, L-Phenylalanine, L-Threonine, Tween 80, α-Cyclodextrin, α-D-Lactose, β-Methyl-D-Glucoside). The effect of origin and season was tested by permutational multivariate analysis based on Euclidean distance using (adonis2 function “vegan”) package ver. 2.6-4 [17], also referred to as adonis PERMANOVA. Both PCA and adonis PERMANOVA analyses were carried out in R environment ver. 4.3.3 [18] (R Core Team, Vienna, Austria, 2024).

Shannon index (*H*) [19] and Simpson (*D*) index were calculated with the equations:

(1) $$H=-\sum pi\times ln\left(pi\right)$$

(2) $$pi=\frac{ai}{\sum ai}$$

(3) $$D=\frac{\sum ai(ai-1)}{A(A-1)}$$

Equations (1) and (2) are the Shannon index, where *pi* is the proportional color development of the well (*ai*) over the total color development of all wells of a technical replica. Equation (3) is the Simpson index where (A) is the total color development of all wells.

## 3. Results

### 3.1. Selected Carbon Sources by Microbial Communities from Both Wild-Caught and Farmed Specimens

Phenotypic differences between wild-caught and farmed seabream from various organs were initially observed. During the 48 h incubation period, all selected metabolites exhibited increased dye development. Out of the four organ sections examined (anterior and posterior gut, cloaca and gills), the anterior and posterior gut samples were excluded from the analysis. This decision was based on the high variability observed within the technical replicates due to challenges in achieving the correct dilutions. Furthermore, the presence of intestinal contents could not be reliably predicted, as both full and empty guts were sampled during the experiment. From a technical standpoint, sampling the anterior and posterior gut also involved complex dissection procedures, while accessing the gills and cloaca was comparatively easier, especially for industrial purposes. Consequently, the analysis focused on the gills and cloaca samples, which proved to be reliable sources.

**Figure 2:**
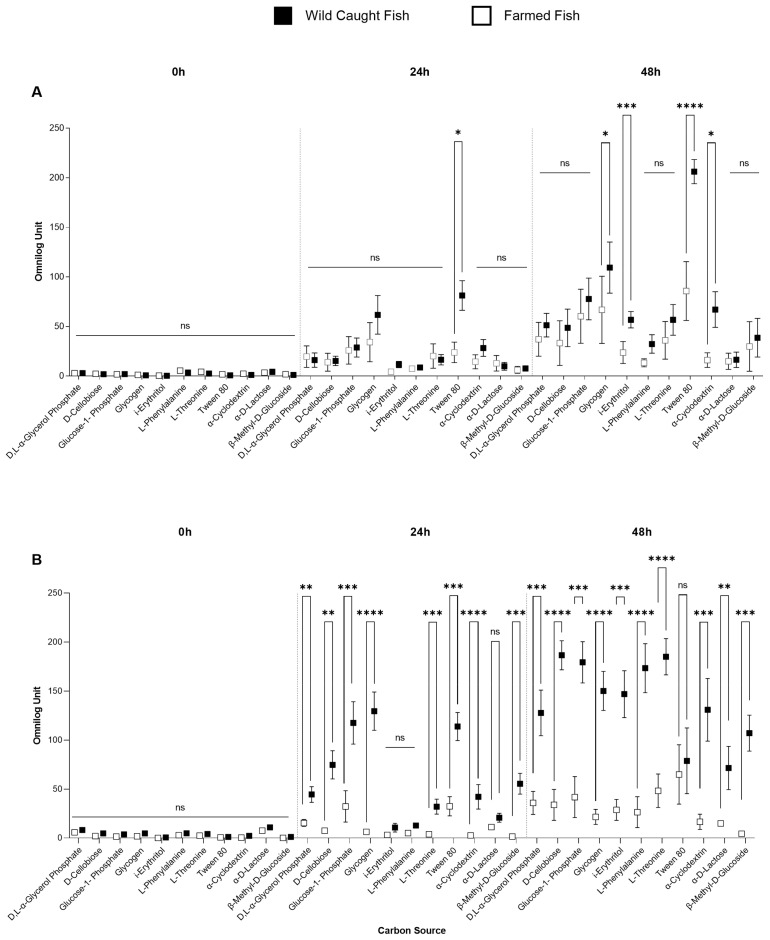
The utilization of selected carbon sources by microbial communities from both wild-caught and farmed specimens was assessed in cloaca (**A**) and gills (**B**), across three distinct time frames. Horizontal bars represent Kruskal–Wallis test, error bars represent standard errors. If error bars are not visible, it is because the size of the symbol (or box) is bigger than the error bars. **** *p* < 0.001; *** 0.0002; ** 0.0021; * 0.0332; ns, not significant.

Microbiota from gills and cloaca were therefore deeply analyzed. To that end, we chose three time points that showed the most significant differences, which were at 0 h, 24 h and 48 h of incubation. Several metabolites, including, *β*-methyl-D-Glucoside, glycogen, D-cellobiose, glucose-1-phosphate and Tween 80, were selected as the most promising metabolites to be monitored. The results of our experiments showed limited differences in Arbitrary OmniLog Units (AOU) between wild-caught and farmed seabream when the cloaca was tested (Figure 2A). However, when the experiment was repeated with the gills, several significant differences were observed for all selected metabolites at both 24 and 48 h (Figure 2B). Furthermore, when we compared the microbial communities obtained from the gills in terms of Arbitrary OmniLog Units at 48 h, we found significant differences between the farmed and wild-caught sorts for the following metabolites: *β*-methyl-D-Glucoside (4.4 ± 1.6 AOU and 107.1 ± 18.9 AOU), *α*-ciclodextrin (16.6 ± 6.7 AOU and 131 ± 18.9 AOU), glycogen (21.6 ± 5.9 AOU and 150.2 ± 13.5 AOU), D-cellobiose (34 ± 10.9 AOU and 186.6 ± 11.8 AOU) and Glucose-1-phosphate (41.8 ± 15.4 AOU and 179.4 ± 16.3 AOU) (Figure 2). We selected these metabolites as they can discriminate between the microbial communities of wild-caught and farmed seabream on the gills. Tween 80 (85.7 ± 18.9 AOU and 206.2 ± 8.7 AOU) was the only metabolite able to show discrimination between wild-caught and farmed fish in samples derived from cloaca, even after 24 h.

### 3.2. Comparison of the Gills and the Cloaca

**Figure 3:**
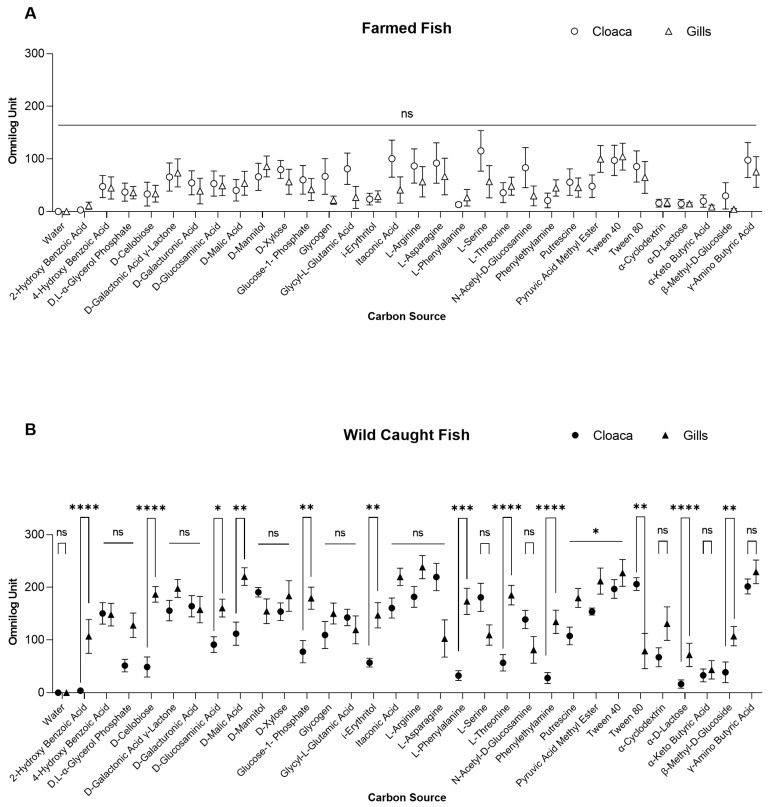
Different carbon source utilization from microbial communities sampled from the gills and cloaca compared at the sampling point of 48 h. Farmed (**A**) and wild-caught (**B**) sources. Horizontal bars represent Kruskal–Wallis test, while error bars represent standard errors. If error bars are not visible, it is because the size of the symbol (or box) is bigger than the error bars. **** *p* < 0.001; *** 0.0002; ** 0.0021; * 0.0332; ns, not significant.

An analysis was also proposed to discriminate the sampling site of the organ, by comparing gills and cloaca sites. The data are presented for both farmed and wild-caught fish (Figure 3). In farmed seabream, no differences in term of AOU were shown among both organs at the time point of 47 h. On the other hand, in wild-caught seabream we selected six metabolites showing the highest differences between gills and cloaca: D-Cellobiose (186.6 ± 11.8 AOU and 48.6 ± 12.4 AOU), L-Phenylalanine (173.4 ± 16.4 AOU and 32.3 ± 6.8 AOU), L-Asparagine (102.7 ± 19.9 AOU and 219.7 ± 14.7 AOU), L-Threonine (185.1 ± 12.3 AOU and 56.7 ± 10 AOU), Phenylethylamine (134.4 ± 16.5 AOU and 27.8 ± 7.1 AOU) and Tween 80 (78.9 ± 18.8 AOU and 206.2 ± 8.7 AOU).

### 3.3. Mock Experiment

**Figure 4:**
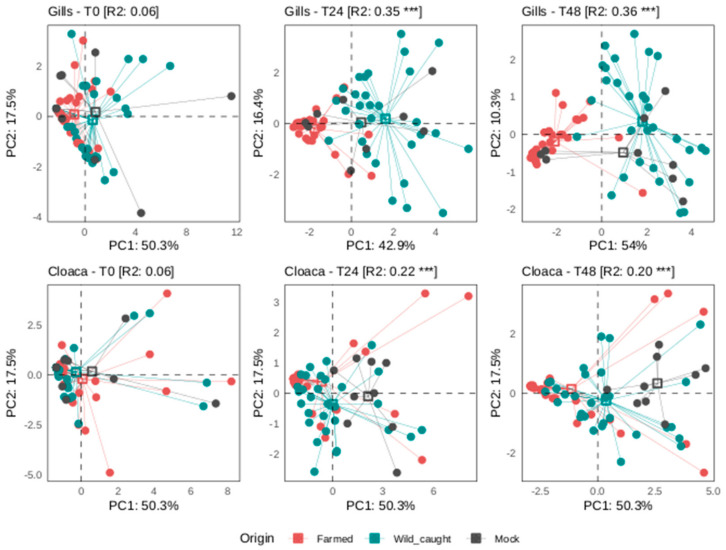
Differences in substrate consumption profiles in samples from different fish origins. Principal Component Analysis (PCA) based on Euclidean distance reporting samples from different fish origins (color scheme). Fish tissue (gills or cloaca) and related time point (T0, T24 and T48) are reported at the top of each panel. R-squared (R2) values and significant effect of origin variable, tested with adonis PERMANOVA, are given in square brackets at the top of each panel. A significant effect is indicated with asterisks (*** *p* < 0.001).

To evaluate the hypothesis that EcoPlate phenomics can aid in identifying traceability, a mock (blind) experiment was conducted. The operator was given unidentified resuspensions of gills and cloaca, which could have originated from either wild-caught or farmed sources. A PCA analysis was performed to reduce the number of variables associated with different sampling times, where variations in OmniLog Units were observed. Subsequently, a PCA analysis was conducted using the timepoints described in the Section 2 and data from the most diverse metabolites in gills and cloaca (Figure 4). PCA based on Euclidean distance showed the samples’ distribution and the expression of the total amount of consumed substrates, grouped according to fish origin but depending on the time point considered (Figure 4). In detail, the distribution of the samples from different fish origins and the mock experiment showed a clear overlap at time T0 in both fish tissue datasets; this evidence was confirmed by adonis PERMANOVA (Gills-T0 and Cloaca-T0 in Figure 4). After 24 and 48 h, a clear separation among samples from different fish origins was evident for both the gill and cloaca datasets including the mock test (Gills-T24 and t48; Cloaca-T24 and T 48 in Figure 4). In particular, the wild-caught group from the gill dataset showed a higher degree of separation compared to the farmed group. Overall, the group separation after 24 and 48 h was more effective in the gills dataset and this was corroborated by the R-squared values (also referred to as explained variance) from adonis PERMANOVA (Figure 4). The multivariate analyses (adonis PERMANOVA) were carried out using a two-factor model formula, adding a seasonal effect (sampling in April 2023 and February 2024, see Section 2) together with the origin variable. As expected, to some extent, the analysis also highlighted a season effect, evident in the cloaca dataset but negligible in the gill dataset. The analysis highlighted a greater influence of season on the microbial communities resident in the cloaca and their effect on the consumption of the different substrates, but this was not the case for those of the gills, corroborating the evidence that the gills represent the ideal tissue for the development of this methodical approach in fish control.

Origin was the main variable able to explain the main differences highlighted in the profiles of the substrates consumed after 24 and 48 h. This was evident for the gills as described above and highlighted by the R-squared values from the multivariate analysis (R-squared from adonis PERMANOVA in Table S1).

**Figure 5:**
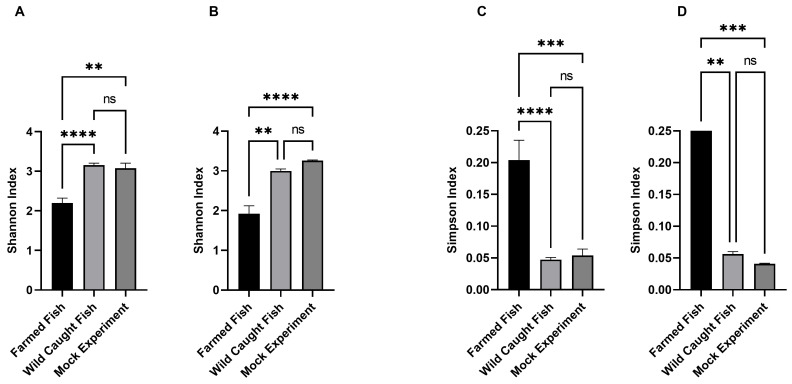
Shannon and Simpson indexes of gills (**A**,**B**) and cloaca (**C**,**D**) from farmed, wild-caught and unknown samples (mock experiment). Horizontal bars represent Kruskal–Wallis test, while error bars represent standard errors; **** *p* < 0.001; *** 0.0002; ** 0.0021; ns, not significant.

In addition, the Shannon and Simpson indexes were used to significantly assess similarities and differences among the gills and cloaca of farmed, wild-caught and unknown samples by including all 31 wells for each replica from gills at 48 h (Figure 5). Interestingly, both indexes showed significant differences for the gills in both farmed and wild-caught sorts, while the mock sample (wild-caught seabreams) was the same with its homolog (Figure 5A,B). Unexpectedly, in the cloaca sample, both indexes also showed significant differences between farmed and wild-caught seabreams, along with the mock samples (Figure 5C,D).

In summary, both the PCA analysis and the Shannon and Simpson indexes in this experiment demonstrated that the EcoPlate approach obtained from the microbial community from gills successfully distinguished between wild-caught and farmed samples. The unknown sample consistently aligned with the wild-caught group, supporting the tracing capabilities of this system.

## 4. Discussion

The study of microbiota as a tracing tool has gained significant attention in recent years due to its crucial role in various aspects of food characterization. Microbiota has been used to trace dairy products [20,21], fruits [22], honey [23], water [24,25], meat [26,27,28] and seafood [29]. These examples involve mainly two techniques: Denaturing Gradient Gel Electrophoresis (DGGE) and Next Generation Sequencing (NGS).

The composition and functioning of microbial communities can also be characterized by using a phenomics approach, developed to characterize and analyze the observable characteristics or phenotypes of a specific microbial community [30].

In this study, we present Biolog EcoPlate as a viable method for assessing traceability, offering additional techniques over the existing DGGE and NGS. The EcoPlate test is not only easy to perform but is also cost-effective, eliminating the need for complex equipment. Even if we used an OmniLog machine for initial high-throughput analysis, this approach needs a simple 96-well plate spectrophotometer. Through our mock experiment, we successfully demonstrated the effectiveness of our method in identifying the origin of a blind sample. By utilizing specific conditions, including the use of gill-derived materials and monitoring OmniLog Units (color development) at 48 h for carbon sources such as D,L-α-Glycerol Phosphate, D-Cellobiose, Glucose-1- Phosphate, Glycogen, i-Erythritol, L-Phenylalanine, L-Threonine, Tween 80, α-Cyclodextrin, α-D-Lactose and β-Methyl-D-Glucoside, we achieved accurate clusterization of the sample’s origin.

The Biolog EcoPlate was developed with a specific focus on community analysis and microbial ecological studies. It was initially designed in response to the needs expressed by a group of microbial ecologists who were utilizing the Biolog GN MicroPlate but desired a panel that offered replicate tests [31]. Further recent analyses have studied reproducibility by sequencing the bacterial communities enriched within each well. Comparisons of alpha and beta diversity in these systems via NGS showed that, while the composition of the communities that grow to inhabit the wells in each substrate array diverges sharply from that of the original community in the inoculum, the final enrichment is dominated by one or several OTUs [32]. Each dominating microbial community is well established and the enrichment is reproducible and stable. The reproducibility of EcoPlate has been shown by other studies [32,33,34]. With reference to the standardization for industrial purposes, we identified specific time points to complete the analysis; however, the observation of a full time-course profile (kinetics of development of the pigment) may give more information than measurements at one or two time points [35].

Several studies have explored the use of microbiota for traceability purposes in differentiating between farmed or wild-caught seafood, including fishing sites. Our parallel research has demonstrated that by analyzing the bacterial V3-V4 region of the 16S rRNA genes, we can effectively profile the diversity of gill bacterial communities in seabass and seabream. This profiling allowed researchers to identify distinctive bacterial signatures correlated with three proximate fishing zones along the Tuscan coast [29]. The use of NGS has revealed that certain bacterial taxa are uniquely tied to their respective fishing area, independent of the fish species. Furthermore, this study reaffirms the suitability of gill tissues for fish-traceability research, showing that the gills’ microbial communities are highly sensitive to environmental changes, thus providing a reliable measure of habitat variations [29,36].

Another study correctly labeled fish samples identified both with taxonomical name and “farmed” or “wild-caught”; the study was conducted using 16S rDNA microbial profiling and next-generation sequencing (NGS) [28]. The study included farmed fish, specifically tilapia and wild-caught fish specimens, such as wild salmon. Total DNA was extracted from the skin mucus of the fish samples via swabbing. The researchers used Illumina MiSeq to sequence the V3-V4 regions of the 16S rDNA that were amplified. The study aimed to assess mislabeling (which also includes “farmed” or “wild-caught”) by analyzing the microbial profiles. The results showed that mislabeling was evident when assessing Faith’s phylogenetic diversity but not with Pielou’s evenness index [28]. Specifically, Antarctic toothfish and Patagonian toothfish were frequently mislabeled [28]. The authors suggested the need to identify specific indicator microorganisms that are more abundant in wild conditions. For example, *Janthinobacterium* was found in higher quantities in the gut of farmed Atlantic salmon in previous studies [28,37], indicating that certain microorganisms may serve as indicators of wild conditions.

Our research demonstrated that certain carbon sources such as D,L-α-Glycerol Phosphate, D-Cellobiose, Glucose-1- Phosphate, Glycogen, i-Erythritol, L-Phenylalanine, L-Threonine, Tween 80, α-Cyclodextrin, α-D-Lactose and β-Methyl-D-Glucoside showed the ability to diversify microbial communities between farmed and wild-caught organisms, specifically in the gills at the 48 h time point. Interestingly, other carbon sources present in the Biolog EcoPlate, such as amines, some amino acids, some carboxylic acids and phenolic compounds, did not exhibit significant differences. The reason behind the higher differentiation observed with these carbon sources remains intriguing. While D-cellobiose, α-cyclodextrin and glycogen are all glucose polymers or dimers, indicating a potential enrichment of chemorganotrophs, we did not investigate the specific microbial species enriched in this experiment. It is important to note that characterizing the most representative enriched species falls outside the scope of this manuscript. We deliberately did not perform NGS in this study to avoid shifting the focus of the take-home message away from the overall phenotypic outcomes and towards a few specific bacteria or fungal species. Future research should prioritize exploring the ecological roles of these carbon sources in microbial community differentiation, potentially through metagenomic analysis, to fully elucidate their impact on the gill microbiome in farmed and wild fish.

In addition, further EcoPlate studies should be conducted to demonstrate that gut and gill microbiomes could reveal past veterinary treatments, for example, past prophylactic uses of antibiotics even in the absence of the chemical detection of antibiotics. Environmental pollution constitutes a major factor reshaping the gut microbiota as well [38]. In addition, although gill microbiota seems to be influenced by host-specific factors, they are indeed strictly connected with the aquatic environment, and may be influenced by the free-living bacterial community [36].

## 5. Conclusions

The physiological profiling of wild-caught seabream has shown distinct patterns when compared to those of farmed counterparts. The use of the Biolog EcoPlate technique was instrumental in differentiating the spatial and temporal microbial community profiles associated with gills. This method is advantageous for industry application due to its simplicity. It requires minimal specialized equipment and technical expertise, requiring only a standard 96-well plate spectrophotometer and low-cost consumables, while also eliminating the need for specialized knowledge in NGS bioinformatics, reducing labor and material costs. Our pilot study effectively validated the method’s capacity to pinpoint the origin of a blind sample, reinforcing its potential utility. The implications of this are significant, given the high market value of wild-caught seabream for both consumers and retailers.

## Data Availability

The original contributions presented in the study are included in the article/Supplementary Materials, further inquiries can be directed to the corresponding author.

## Supplementary Materials

The following supporting information can be downloaded at: https://www.mdpi.com/article/10.3390/foods13172726/s1, Table S1: Results from adonis PERMANOVA.

## References

[1] Islam S., Cullen J.M. (2021). Food Traceability: A Generic Theoretical Framework. Food Control.

[2] EU Eurobarometer. https://europa.eu/eurobarometer.

[3] (2020). The EU Agri-Food Fraud Network and the Administrative Assistance and Cooperation System, Annual Report. https://food.ec.europa.eu/safety/acn/reports-and-publications_en.

[4] Donnelly K.A.M., Olsen P. (2012). Catch to Landing Traceability and the Effects of Implementation—A Case Study from the Norwegian White Fish Sector. Food Control.

[5] Pereira L.A., Santos R.V., Hauser M., Duponchelle F., Carvajal F., Pecheyran C., Bérail S., Pouilly M. (2019). Commercial Traceability of Arapaima Spp. Fisheries in the Amazon Basin: Can Biogeochemical Tags Be Useful?. Biogeosciences.

[6] Tulli F., Moreno-Rojas J.M., Messina C.M., Trocino A., Xiccato G., Muñoz-Redondo J.M., Santulli A., Tibaldi E. (2020). The Use of Stable Isotope Ratio Analysis to Trace European Sea Bass (*D. labrax*) Originating from Different Farming Systems. Animals.

[7] Pascual-García A., Bell T. (2020). Community-Level Signatures of Ecological Succession in Natural Bacterial Communities. Nat. Commun..

[8] Faust K., Bauchinger F., Laroche B., de Buyl S., Lahti L., Washburne A.D., Gonze D., Widder S. (2018). Signatures of Ecological Processes in Microbial Community Time Series. Microbiome.

[9] Callao M.P., Ruisánchez I. (2018). An Overview of Multivariate Qualitative Methods for Food Fraud Detection. Food Control.

[10] Cristina Dos Santos R., Fokar M., Romagnoli E.M., Aziz M., Bento J.M.S., Paré P.W. (2021). Monitoring a Beneficial Bacterium (Bacillus Amyloliquefaciens) in the Rhizosphere with Arugula Herbivory. Rhizosphere.

[11] González M.-L., Fuentes M.E., Molina V., Quiñones R.A. (2023). Effects of Ethoxyquin on Metabolism and Composition of Active Marine Microbial Communities. Aquaculture.

[12] Niu S., Zhang K., Li Z., Xie J., Wang G., Li H., Yu E., Xia Y., Tian J., Gong W. (2023). Analysis of the Structure and Function of Microbial Community in Late-Stage of Grass Carp (*Ctenopharyngodon idella*) Farming Ponds. Aquac. Rep..

[13] Sas-Paszt L., Trzciński P., Lisek A., Gluszek S., Matysiak B., Kaniszewski S. (2023). The Influence of Consortia of Beneficial Microorganisms on the Growth and Yield of Aquaponically Grown Romaine Lettuce. Agronomy.

[14] Griffiths B.S., Ritz K., Wheatley R.E. (1997). Relationship between Functional Diversity and Genetic Diversity in Complex Microbial Communities. Microbial Communities.

[15] Regione Toscana L.R. N. 66/2005 Programma Annuale Pesca Professionale e Acquacoltura 2006. https://www.regione.toscana.it/documents/10180/70946/programma_pesca_2006/21d1061a-8360-416e-b0f7-b668532e8891.

[16] Council Regulation (EC) (1995). No. 2597/95 of 23 October 1995 on the Submission of Nominal Catch Statistics by Member States Fishing in Certain Areas Other than Those of the North Atlantic. Official J..

[17] (2024). Community Ecology Package [R Package Vegan Version 2.6-6.1].

[18] R Core Team R. (2024). A Language and Environment for Statistical Computing.

[19] Galieva G.S., Gilmutdinova I.M., Fomin V.P., Selivanovskaya S.Y., Galitskaya P.Y. (2018). Monitoring Soil Bacteria with Community-Level Physiological Profiles Using Biolog^TM^ ECO-Plates in the Republic of Tatarstan (Russia). IOP Conf. Ser. Earth Environ. Sci..

[20] Bonizzi I., Feligini M., Aleandri R., Enne G. (2007). Genetic Traceability of the Geographical Origin of Typical Italian Water Buffalo Mozzarella Cheese: A Preliminary Approach. J. Appl. Microbiol..

[21] Feligini M., Panelli S., Sacchi R., Ghitti M., Capelli E. (2015). Tracing the Origin of Raw Milk from Farm by Using Automated Ribosomal Intergenic Spacer Analysis (ARISA) Fingerprinting of Microbiota. Food Control.

[22] El Shobaky A., Meile J.-C., Montet D. (2015). New Traceability Strategies Based on a Biological Bar Code by PCR-DGGE Using Bacterial and Yeast Communities for Determining Farming Type of Peach. Egypt. J. Basic Appl. Sci..

[23] Kafantaris I., Amoutzias G.D., Mossialos D. (2021). Foodomics in Bee Product Research: A Systematic Literature Review. Eur. Food Res. Technol..

[24] da Silva Costa R., Sainara Maia Fernandes T., de Sousa Almeida E., Tomé Oliveira J., Carvalho Guedes J.A., Julião Zocolo G., Wagner de Sousa F., do Nascimento R.F. (2021). Potential Risk of BPA and Phthalates in Commercial Water Bottles: A Minireview. J. Water Health.

[25] Sala-Comorera L., Blanch A.R., Casanovas-Massana A., Monleón-Getino A., García-Aljaro C. (2019). Traceability of Different Brands of Bottled Mineral Water during Shelf Life, Using PCR-DGGE and next Generation Sequencing Techniques. Food Microbiol..

[26] Cauchie E., Delhalle L., Taminiau B., Tahiri A., Korsak N., Burteau S., Fall P.A., Farnir F., Baré G., Daube G. (2020). Assessment of Spoilage Bacterial Communities in Food Wrap and Modified Atmospheres-Packed Minced Pork Meat Samples by 16S RDNA Metagenetic Analysis. Front. Microbiol..

[27] Koo O.-K., Baker C.A., Kim H.-J., Park S.H., Ricke S.C. (2016). Metagenomic Assessment of the Microbial Diversity in Ground Pork Products from Markets in the North Central Region of South Korea. J. Environ. Sci. Health Part B.

[28] Shehata H.R., Mitterboeck T.F., Hanner R. (2020). Characterization of the Microbiota of Commercially Traded Finfish Fillets. Food Res. Int..

[29] Meriggi N., Russo A., Renzi S., Cerasuolo B., Nerini M., Ugolini A., Marvasi M., Cavalieri D. (2024). Enhancing Seafood Traceability: Tracking the Origin of Seabass and Seabream from the Tuscan Coast Area by the Analysis of the Gill Bacterial Communities. Anim. Microbiome.

[30] Sofo A., Ricciuti P. (2019). A Standardized Method for Estimating the Functional Diversity of Soil Bacterial Community by Biolog^®^ EcoPlates Assay—The Case Study of a Sustainable Olive Orchard. Appl. Sci..

[31] Insam H. (1997). A New Set of Substrates Proposed for Community Characterization in Environmental Samples. Microbial Communities.

[32] Flynn T.M., Koval J.C., Greenwald S.M., Owens S.M., Kemner K.M., Antonopoulos D.A. (2017). Parallelized, Aerobic, Single Carbon-Source Enrichments from Different Natural Environments Contain Divergent Microbial Communities. Front. Microbiol..

[33] Haack S.K., Garchow H., Klug M.J., Forney L.J. (1995). Analysis of Factors Affecting the Accuracy, Reproducibility, and Interpretation of Microbial Community Carbon Source Utilization Patterns. Appl. Environ. Microbiol..

[34] Rutgers M., Wouterse M., Drost S.M., Breure A.M., Mulder C., Stone D., Creamer R.E., Winding A., Bloem J. (2016). Monitoring Soil Bacteria with Community-Level Physiological Profiles Using Biolog^TM^ ECO-Plates in the Netherlands and Europe. Appl. Soil Ecol..

[35] Hackett C.A., Griffiths B.S. (1997). Statistical Analysis of the Time-Course of Biolog Substrate Utilization. J. Microbiol. Methods.

[36] Pratte Z.A., Besson M., Hollman R.D., Stewart F.J. (2018). The Gills of Reef Fish Support a Distinct Microbiome Influenced by Host-Specific Factors. Appl. Environ. Microbiol..

[37] Gajardo K., Rodiles A., Kortner T.M., Krogdahl Å., Bakke A.M., Merrifield D.L., Sørum H. (2016). A High-Resolution Map of the Gut Microbiota in Atlantic Salmon (*Salmo salar*): A Basis for Comparative Gut Microbial Research. Sci. Rep..

[38] Evariste L., Barret M., Mottier A., Mouchet F., Gauthier L., Pinelli E. (2019). Gut Microbiota of Aquatic Organisms: A Key Endpoint for Ecotoxicological Studies. Environ. Pollut..

