# Supplementary material for: A Microbial Phenomics Approach to Determine Metabolic Signatures to Enhance Seabream Sparus aurata Traceability, Differentiating between Wild-Caught and Farmed
Source: Foods. 2024 Aug 28;13(17):2726. doi: 10.3390/foods13172726 (PMC11394949; doi:10.3390/foods13172726)
Supplement: Supplementary file 1 [file foods-13-02726-s001.zip › foods-3113747-supplementary.pdf]

Table S1. Results from adonis PERMANOVA.

Tissue	Time	Factor	R-squared	Sign
Gills	T0	Origin	0.06	
		Season	0.01	
	T24	Origin	0.35	***
		Season	0.03	*
	T48	Origin	0.36	***
		Season	0.06	***
Cloaca	T0	Origin	0.02	
		Season	0.18	***
	T24	Origin	0.22	***
		Season	0.17	***
	T48	Origin	0.2	***
		Season	0.21	***

The table reports the results from multivariate analysis adonis PERMANOVA performed on euclidean distance -based dataset (see materials and metods for additional details). The factors tested in the model formula are the Origin and Season. The statistically signifcant effect of the factor tested are highlited using asterisks (*, $p<0.05$; ***, $p<0.001$).
